# The Naïve nurse: revisiting vulnerability for nursing

**DOI:** 10.1186/1472-6955-11-5

**Published:** 2012-04-20

**Authors:** Laura Tomm-Bonde

**Affiliations:** 1 Department of Nursing, University of Victoria, Finnerty Road, Victoria, BC, Canada

## Abstract

**Background:**

Nurses in the Western world have given considerable attention to the concept of vulnerability in recent decades. However, nurses have tended to view vulnerability from an individualistic perspective, and have rarely taken into account structural or collective dimensions of the concept. As the need grows for health workers to engage in the global health agenda, nurses must broaden earlier works on vulnerability, noting that conventional conceptualizations and practical applications on the notion of vulnerability warrant extension to include more collective conceptualizations thereby making a more complete understanding of vulnerability in nursing discourse.

**Discussion:**

The purpose of this paper is to examine nursing contributions to the concept of vulnerability and consider how a broader perspective that includes socio-political dimensions may assist nurses to reach beyond the immediate milieu of the patient into the dominant social, political, and economic structures that produce and sustain vulnerability.

**Summary:**

By broadening nurse’s conceptualization of vulnerability, nurses can obtain the consciousness needed to move beyond a peripheral role of nursing that has been dominantly situated within institutional settings to contribute in the larger arena of social, economic, political and global affairs.

## Background

Nurses claim vulnerability as a central and important concept within nursing [1]. There have been countless studies that have identified the concept of vulnerability as a key factor in determining health status of individuals, groups and communities [2-8]. With few exceptions, however, nurse theorist have not elaborated upon the concept of vulnerability, resulting in an underdeveloped concept [3] that is narrowly defined, lacking “an archaeology of the social, political and economic” [9] dimensions that produced ideologies and sustain certain structures that determine health. Nurse scholars tend to view vulnerability as a source of stimuli to which individuals respond or as the interactional arena in which individual’s accommodate, assimilate, or adjust to the prevailing social customs and expectations of society’s dominant ideology.

With a tendency to concentrate almost exclusively upon the adaptive capacity of individuals, nursing theories do not encompass explanations for persons, groups or populations who refuse to reject accommodation to vulnerability that present intolerable or unacceptable social, political or economic conditions. There are contexts where vulnerability has worked to incite social or political movements. Within these contexts, there are people who refuse to accept perceived injustices or who refuse to adjust or adapt to the societies dominant ideologies. Nursing theories lack substantive explanations for contexts where vulnerability is deeply rooted and social movements arise whereby the structure of society is challenged. Examples of such movements include the women suffrage, labour, liberation and anti-racism movements.

Although nurses are aware of the root causes of person’s vulnerability that may cause or potentiate health problems, they often approach these origins of vulnerability from epidemiological perspectives [10]. That is, the patient is examined for causative agents that underlie the health issue. Factors such as toxic agents, microbes, viruses or other potential health hazards are considered as acting on the individual and threatening an individual’s health and well-being [10]. Chopoorian [10] argues that the concept of environment is not analyzed as social landscape, as geography, and lacks the archaeology of the social, political, and economic worlds that influence clients states and nursing roles. It is my view that the concept of vulnerability can be housed in Chopoorian’s view of environment.

While the concept of vulnerability is given attention in nursing, it is the concept of the person that holds the paramount place in the conceptualization and therefore I argue in the same vein as Chopoorian, that the concept of vulnerability within nursing lacks consciousness in the larger arena of social, economic and political affairs. As a result, nurses have not been provided with the framework to view vulnerability from a social, economic or political lens and consequently do not regularly lobby or otherwise participate in local, national or international political offices, such as participate in policy formation, or sit on committees/ boards that address policy in relation to these levels. Yet, nurses have frontline experience to the most detrimental effects of vulnerability on the fate of particular individuals, groups and populations.

It was not until I began to work as a nurse in Mozambique within the international aid community that I came to realize my own deficiency of understanding how social, economic and political structural factors intersect and shape people’s ability to be healthy. Consequently, I was a naïve nurse, lacking the knowledge and skill on how to engage with the concept of vulnerability at a practice level within a context of widespread vulnerability. At first I engaged with vulnerability through the lens of individualism, which has been influenced by basic principles of neoliberalism, the dominant paradigm guiding both the West and international development policy today [11]. This naïve view of vulnerability clashed with my on-the-ground experience in Mozambique where vulnerability is widespread, directly resulting from historical, social, economic and political dimensions, rending whole populations vulne-rable with dominant power-structures acting as barriers rather than supportive openings where people are able to access supports to overcome their own state of affairs. For this reason, I realized the need for nursing to expand its view of vulnerability and challenge the current conceptualizations of vulnerability in nursing.

On this basis I seek demonstrate how the concept of vulnerability in nursing has been shaped within two basic premises of a Western-based neoliberal paradigm, namely: individualism and economic rationality. The purpose of this paper is threefold: (a) to review the origins of the term vulnerability; (b) to explore how vulnerability has been constructed within nursing and influenced by a Western-based neoliberal paradigm; and (c) to engage in a dialogue about the potential hazards of maintaining a view of vulnerability in nursing confined to an individualism paradigm. These hazards include, but are not limited to, re-colonizing practices that ignore structural factors that impede people’s ability to make rational choices, undermining meaningful efforts to promote social justice within a global context. Finally, I will offer suggestions as to how we as nurses might broaden our view of vulnerability in order to transcend our Western-based analysis and change our practice both within and beyond our Western borders, with the hope that this knowledge will assist other nurses from being, like myself, the naïve nurse.

## Discussion

### The current status of the concept of vulnerability

Nurses in the West have given considerable attention to the concept of vulnerability in recent decades. It has become a “trendy concept” [1] without specific meaning and for this reason has also been criticised as too loosely defined. Nurses have tended to view vulnerability from an individualistic perspective, and have rarely taken into account social, political and economic dimensions of the concept that define the structural context for which vulnerability emerges. Rather, conceptualizations of vulnerability in nursing have remained within a neoliberal discourse. They have tended to focus on characteristics of individuals and their immediate milieu, rather then reframing the problem within broader environmental and ideological factors that examine the collective dimensions and structural influences of the concept. Collective dimensions can be defined as encompassing a single cultural community group in a territory over which they exercise sovereignty [12]. Collective dimensions of vulnerability are shared experiences of vulnerability by one group; this shared experience by a group is reinforced by political and structural conditions that reinforce a collective experience and norm. Browne [13], a critical nursing scholar, suggests that by uncovering and interrogating the assumptions underlying the dominant neoliberal paradigm in nursing, “new forms of critically oriented knowledge can be generated to address larger structural conditions that contribute to inequities in health and health-care” [13]. The discipline of nursing claims a profound commitment to social justice as a fundamental principle, and therefore understanding ways in which inequities are reinforced is a central task [1,14]. However, uncovering those understandings can be uncomfortable for those who prefer that science be treated as an apolitical enterprise [13].

I hold the view that all forms of knowledge and social engagement cannot be free from the historical, political, cultural and economic forces that shape philosophies, science and knowledge development. As a social constructionist, I hold the view that science cannot be value-free or neutral, but is infused with our perceptions of the world. As a feminist and critical scholar, I hold the view that power-inequities are constantly at play in every social situation. Browne [13] explains that new racisms are produced which draw upon notions of cultural differences and incompatibilities to explain health, social and economic inequities [13]. What is too often ignored, and therefore absent, in much of the nursing literature is an examination of how gender, racialization, class and history intersect to shape socio-political-economic realities and, therefore, health. The nursing literature, particularly in North America, is constrained, failing to examine the political and ideological underpinnings of vulnerability. In its current state, the concept of vulnerability is a rigid, static concept, which does not inform the nursing paradigm in a substantive manner, nor does it foster comprehensive images and understandings of vulnerability. This leads to a narrow view of the concept of vulnerability and language is missing from the literature of nursing theorists to fully capture a comprehensive understanding of vulnerability. Therefore, vulnerability becomes a taken for granted concept, ambiguous, diffuse and blurred, despite the continuous references to it in the language of nursing.

While the conceptualization of vulnerability is given attention, it is largely given this attention at the level of the person, whereby describing vulnerability in relation to the individual’s response to health or illness provoking situations. For this reason, nursing practice is constructed upon assumptions about the patient as a person, family, or community in that interventions are planned for individuals [10]. Nursing practice is concentrated on responding to situations and problems that need to be ameliorated. Nurses become problem solvers at the individual level, without attending to or acting on social, political or economic conditions in the larger society that produce and sustain the individual’s situation. Nurses therefore do not generally “consider strategies for changing, adjusting, or altering environments”, rather it is the individual who must adjust, assimilate, or accommodate to the vulnerable situation and it is the nurses perceived role to support them in this process [10]. Therefore, nursing practice from this narrow perspective perpetuates the idea that the responsibility lies with the individual and little thought is given to addressing societal structures or institutions, the focus of change or adaption remains at the person. A first step to overcoming this constrained discourse on vulnerability is to examine historical and contemporary understandings of vulnerability within the parameters of nursing.

### Etymology of vulnerability

The term vulnerable originates from the Latin term “*vulnus”,* translated as wound. The etymology of this word signifies one’s capability to wound, inflict physical trauma, or the susceptibility to be wounded, that is to experience physical trauma [15]. This suggests that both the victim and the aggressor are equally susceptible to injury of some kind. The Cambridge International Dictionary of English (1995) widens the definition to include psychological hurt [16]. The modern understanding of vulnerability, in addition to physical trauma, includes the human potential to experience psychological harm, spiritual threat, and moral distress [15]. These dictionary definitions are based on a Cartesian view of humans. They separate vulnerability into types: Physical and mental. This is often referred to as Cartesian dualism [17]. Simply, these definitions unveil the semantic biases, Eurocentric binary distinctions and ideological interests within the English language. It is undeniably obvious the Cartesian mental-physical divide that dominates these definitions and ignores a corporal reality constructed through an agenda of power and social order.

Vulnerability is simply understood in terms of a binary between physicality and mentality, without any consideration of sociocultural, socioeconomic or socio-political discursive constructions of vulnerability. Pfeifer [17] points out that language can act as a vehicle of ideology and traps us in strongholds of semantic biases and ideological interests. It is therefore imperative to unveil these semantic biases and ideological interests to break through normally impassable discursive strongholds which can lead to broader understandings of the world, and in this case, broader understandings of the concept vulnerability.

### Consequences of a taken for granted, blurred and ambiguous concept of vulnerability

This paramount focus upon the individual or group of individuals is hard to fault, particularly in contrast to other health professionals, who tend to carve up the person and tackle parts or pieces. Yet there are consequences for the nursing profession as a result of this concentrated attention on persons and tendency to explain the human condition from the immediate surrounding social world of the individual. Nurse’s arena for intervention and action then becomes restricted or limited to institutionally confined settings. Interventions become sandwich into one model, the Westernized-generated biomedical model.

In this paper I analyzed the literature from 1980 to present and searched for articles that attended to the concept of vulnerability. I used the search engines CINAHL, SUMMONS, and google scholar to search for articles. Key words used were vulnerable, vulnerability, nursing, disenfranchised and marginalized. Over 37, 000 hits to the search on the key words ‘vulnerability and nursing’ was generated. Limiting my search to exclude newspaper articles and only scholarly and peered reviewed articles decreased my hits to around 22,000. Further limiting my search to the subject area of nursing got me down to 3,000 articles. From here I began to search through these articles to find ones that were relevant for this paper. What I found was that the articles could be grouped into two main categories, all of which are based from a perspective of individualism. These two categories are as follows: (a) Power and (b) risk. Both of these categories assumed an individualistic approach to vulnerability.

#### Power

Power is a central theme in many articles and nurses have used the concept of power to theorize about and study vulnerability [2,4,18-20]. Within the category of power, authors tended to look at the concept of power in relationship to patients or within the field of nursing work [21] and how these situations create vulnerability. Other authors [22,23] focus specifically on overcoming vulnerability that is produced within a nurse-patient relationship whereby power dynamics influence a state of vulnerability. It is likely that addressing the issue of power stemmed from an increasing push in the late 1980s and early 1990s for healthcare institutions to promote health education within their settings. Nurses were the obvious choice to deliver this education. During the 1980s and 1990s the focus in healthcare was on changing individual behaviour with regards to lifestyle and this meant engaging with patients both on an individual and population level. The strategic approach, in light of this paradigm, was to focus on social responsibility and personal choice [24]. As nurses became the primary deliverer of health promotion/health education messages, nurses began to recognize the fragility in the nurse-patient relationship and recognized there were power dynamics operating and consequently affecting the relationship between the nurse and the patient. A proliferation of literature emerged that focused on vulnerability and power [3,4,18-20,25-29]. Articles generally did not clearly define vulnerability, yet imply that vulnerability had to do with a lack of advocacy and referred to specific populations such as disenfranchised or marginalized populations who were particularly susceptible to vulnerability because they lacked the ability to control situations [2,4,19].

Powerlessness stemming from a number of competing factors plaguing a patient’s locus of control foster vulnerability and the nurse’s role, according to some authors [2,19], was to understand this problem and remedy it in a protectionist way.

The asymmetries of power inequality nurture and sustain vulnerability [18,22,23]. As a result the power asymmetry between the nurse and the patient can have consequential effects on trust. Power imbalances between nurse researchers and patients who are categorized as vulnerable also have ethical implications and the need to acknowledge these in order to protect individuals was noted as important [30]. For nursing scholars who approached the concept of vulnerability from this perspective believed the paramount approach is for nurses to engage in a process of understanding one’s own vulnerability in the relationship (nurse-patient) in order to enter into a power-with versus a power-over. Daniel [22] coins this “mutual vulnerability”. Mutual vulnerability means to balance power by understanding one’s own vulnerability in relationship to the patient. Attending to power and the issues of power that happen for nurses or patients or between nurses and their patients was a dominant approach to the concept of vulnerably in nursing.

As nursing has tended to frame the concept of vulnerability within the context of power imbalance, they have limited their analysis and theoretical discussions to dualistic conceptualizations of vulnerability (nurse-patient/ physician-nurse; researcher-researched). This narrow and limited model demonstrates how nursing in the West has been overwhelmingly influenced by an individualistic paradigm, which negates the examination of vulnerability in the context of broader structural determinations such as the multiple intersections of economic, political and sociocultural spheres that influence and sustain vulnerability. Nursing is therefore ill-equipped to understand the ways in which structural power and the intersections of various identities might influence vulnerability, since nurses do not examine this in their scholarship. In this way, nurses in the West privilege neoliberal principles of individualism rather than attempting to go beyond this dominant paradigm and include collectivist ideas on vulnerability.

#### Risk

This discourse of risk has been maintained as the foundation upon which recent discussions of vulnerability are based [2,8,18,20,21,31-38]. The establishment of a risk paradigm refers to a susceptibility or predisposition that individuals or populations have to harm [19,35,39]. Irurita [19] makes the connection between vulnerability and risk when she describes vulnerability as “being susceptible to physical and or emotional hurt, harm or injury, [or being] defenceless or weak in relation to self protection, open to assault” (p. 11). Vulnerability, in this sense, is presented in terms of susceptibility or in other words, *risk*.

Nursing scholarship has tended to distinctly focus on understanding vulnerability from within the confines of an individual’s experience or situation, which marks the risk paradigm in practice. The emphasis on individual experience within the nursing discipline reflects nursing’s tendency towards epistemological individualism as a theory of knowledge. Brown [13]describes how individualism has served as a fundamental link between liberalism and empiricism. “Epistemological individualism continues to shape contemporary objectivist and postpositivists traditions, particularly in the human sciences” (p. 121). The consequences of addressing the concept of vulnerability within the confines of epistemological individualism in nursing have resulted in narrow and blurred understandings of the concept.

It is this tendency to concentrate almost exclusively upon individual’s adaptive capacities and how individuals adapt to their immediate surroundings that become the central focus of understanding the concept of vulnerability. There is little attention given in nursing scholarship to broadening nurses understanding of vulnerability to incorporate how the environment or context in which vulnerability occurs shapes and sustains vulnerability. The structural dimensions have been overlooked.

The social, political and economic worlds that influence both client states and nursing roles are absent from the literature. As a result, nursing lacks “an archaeology” [10] of the social, political and economic dimensions that can inform their practice. In some ways, nursing is paralyzed within an individualist paradigm, lacking the conceptual tools to look beyond the person’s immediate surroundings that might help produce explanations for persons or groups who refuse to reject accommodation to vulnerability that present intolerable circumstances like those in developing countries where that are mass situations of poverty. Allowing nursing to reach beyond the immediate milieu of the patient into the dominant social, political and economic structures that produce behaviours associated with class relationships, power relations, political interests, economic policies and ideologies such as sexism, racism, ageism, and classism that influence persons in their worlds is a fundamental step in understanding the complexities that interfere with health and eventually cause illness. It is in this vein that I argue for a broader conceptualization of the concept of vulnerability that will enable nurses to move the concept of vulnerability into the twenty-first century.

## Summary

### Moving the concept of vulnerability in nursing into the twenty-first century

The purpose of this paper is to revisit nursing contributions to the concept of vulnerability and consider how a broader perspective that includes socio-political dimensions may help to prevent a blurred understanding of vulnerability in nursing. Perspectives on vulnerability in nursing have tended to be governed by neoliberal principles that focus on individualism, rather than collective perspectives that make explicit the societal or community vulnerability, political, economic and structural vulnerabilities and intersecting identities that render people vulnerable such as class, race, and gender. It is evident that health discourses shape nursing practice in many ways and “in order to advance nursing science toward socially progressive praxis, critiques of our own complacency with the ‘ruling relations’ as they are enacted in research, theory, practice and education are required” [13].

Neoliberalism emerged in Western society over the past twenty years, as the dominant governing ideology that concentrates on individuals’ responsibility for themselves by promoting “self-sufficiency” [40] as a core value. Likewise, this ideology encourages people to believe that big government is bad, thus, shifting responsibility for social welfare away from government and onto communities and individuals [40]. Conservative and liberal governments have successfully packaged the neoliberal ideology as a positive upstream approach to social governing.

This dominant paradigm influenced many countries to embrace lifestyle approaches to individual health as the way to address broad health issues. For example, behaviour change campaigns that focus on eating healthy food, taking in less salt, and exercising as means to maintain health have become mainstream approaches in health promotion education across North America. It is not surprising therefore, that vulnerability was conceptualized at the individual level rather than within the broader societal and structural sphere.

There are consequences, however, for the nursing profession as a result of this concentrated attention on persons and tendency to explain the human condition from the immediate surrounding social world of the individual. As noted earlier in this paper, Chopoorian [10] explains that nursing’s arena for intervention and action has been restricted or limited to institutionally confined settings. As well, nurse’s role as largely institutionally confined limits them to supporting patients in their vulnerable states rather than broadening their influence on the development of national policy, foreign policy, labour policy, economic affairs, international peace efforts, and the like. The societal structures and the human, social relationships that result from the internal dynamics of these structures are process that influence health or illness states, and although nurses have first hand knowledge of health and illness, they struggle for a substantive place in the formulation of social, political and economic policies related to health. They remain cemented to a paradigm for nursing intervention that is individualized and situationally and institutionally orientated [10].

Chopoorian [10] was the first to suggest a broader conceptualization of the concept of environment and later Stevens [9] builds on Chopoorian’s work and offers up critical social theory as one approach to broadening nursing theory in relation to environment. In a similar fashion, Nichiata, Bertolozzi, Takahashi and Fracolli [41], nursing scholars based in Latin America, suggest that we can understand vulnerability as a ‘collective dimension’ of health [41] and recommend nursing scholarship in relationship to vulnerability draw on critical social theory to understand the concept more broadly. Nichiata et al. contend that the focus on vulnerability in nursing is still narrow [41]. Knowledge generation that focuses on social issues more critically will likely assist nurses in grappling with global health issues often perpetuated by the economic and political situations in countries such as those labeled Third World/North–South Divide/Two-third-One-third. Novel conceptualizations of vulnerability warrants clear delineations and explications in the philosophic, theoretical, research, ethical and practice posture of nursing that draws from critical social theory as suggested by the above authors [9,10,41].

Contemporary authors such as Bjornsdottir [42], Flaskerud et al. [6], Flaskerud and Winslow [8,32], Foth [43], and Higgins, Hoffman and Dworkin [20] are offering promising shifts in nursing’s understanding of vulnerability. They push nurses to consider larger issues influencing vulnerability that go beyond an allegiance to individualism toward broader collective and structural dimensions of vulnerability. This is a significant milestone for nursing as it begins to shift nursing outside a philosophical standpoint of individualism that is rooted in Western scientific thought and urges nurses to imagine other possible perspectives for the advancement of the nursing profession.

## Conclusion

Nurses in the Western world have given considerable attention to the concept of vulnerability in recent decades. This paper has demonstrated how nurses tend to favour the concept of vulnerability from a philosophical perspective of individualism governed and reinforced by the dominant paradigm of neoliberalism. Nurses have failed to examine how this dominance has contributed to a conceptualized version of vulnerability that is irrelevant for the rest of the world that is beyond the Westernized borders. Reviewing the way in which nursing has conceptualized vulnerability in recent decades demonstrates that nurses need to reconsider how current understandings of vulnerability are limited and narrow. This limited, narrow and blurred conceptualization of vulnerability within the nursing literature limits nursing practice to intervening at the level of the person and not beyond. If nurses continue to stay contained within the philosophy of individualism they too will continue to enact the nursing role at the individual level, the privatized level and the institutional level. Nurses will be unable to contribute substantively to issues related to vulnerability that involve the socio-political, socio-economic or socio-cultural aspects of the social world that produce conditions and relationships that influence health of illness states. Nurses need to explicitly apply critical perspectives to the concept of vulnerability so that a more holistic understanding of vulnerability is formed for the nursing discipline. I believe that by doing this nursing educators will be more prepared to teach about vulnerability that interrogates structural components of the concept and envisions collectivist strategies to address problems such as vulnerability in contexts that go beyond the Western borders. As well, opening up an analysis of vulnerability and other concepts as well within nursing can assist nursing in overcoming their stagnant roles that have historically been confined to institutional settings limiting nursing practice roles. By moving beyond individualism, nurses will be more equipped to engage with the global health community more critically and new forms of critically orientated knowledge can be generated to address the larger structural conditions that contribute to vulnerability in health and in health care, which is especially fragile in developing countries such as Mozambique.

## Competing interests

The author declare that they have no competing interests.

## Authors’ contributions

*Laura N. Tomm-Bonde* is a PhD student in the School of Nursing at the University of Victoria. She is interested in international development, global health, North–south partnership and how the HIV/AIDS social situation in Mozambique has been understood. Her education has been supported through a Doctoral Award in the Area of Health Services/Population Health, HIV/AIDS Research from the Canadian Institutes of Health Research.

## Pre-publication history

The pre-publication history for this paper can be accessed here:

http://www.biomedcentral.com/1472-6955/11/5/prepub
